# The role of the surfaces in the photon absorption in Ge nanoclusters embedded in silica

**DOI:** 10.1186/1556-276X-6-135

**Published:** 2011-02-11

**Authors:** Salvatore Cosentino, Salvatore Mirabella, Maria Miritello, Giuseppe Nicotra, Roberto Lo Savio, Francesca Simone, Corrado Spinella, Antonio Terrasi

**Affiliations:** 1 MATIS-IMM-CNR and Dipartimento di Fisica e Astronomia, Università di Catania, Via Santa Sofia 64, 95123 Catania, Italy; 2 IMM-CNR, VIII Strada 5, 95121 Catania, Italy

## Abstract

The usage of semiconductor nanostructures is highly promising for boosting the energy conversion efficiency in photovoltaics technology, but still some of the underlying mechanisms are not well understood at the nanoscale length. Ge quantum dots (QDs) should have a larger absorption and a more efficient quantum confinement effect than Si ones, thus they are good candidate for third-generation solar cells. In this work, Ge QDs embedded in silica matrix have been synthesized through magnetron sputtering deposition and annealing up to 800°C. The thermal evolution of the QD size (2 to 10 nm) has been followed by transmission electron microscopy and X-ray diffraction techniques, evidencing an Ostwald ripening mechanism with a concomitant amorphous-crystalline transition. The optical absorption of Ge nanoclusters has been measured by spectrophotometry analyses, evidencing an optical bandgap of 1.6 eV, unexpectedly independent of the QDs size or of the solid phase (amorphous or crystalline). A simple modeling, based on the Tauc law, shows that the photon absorption has a much larger extent in smaller Ge QDs, being related to the surface extent rather than to the volume. These data are presented and discussed also considering the outcomes for application of Ge nanostructures in photovoltaics.

PACS: 81.07.Ta; 78.67.Hc; 68.65.-k

## Introduction

Nanostructured materials represent a promising route of development for photovoltaics (PV) because of the unique optical and electronic properties caused by the quantum confinement of electrons and holes, allowing to increase the efficiency of the sunlight-electricity conversion [1-8]. It has been argued that quantum dots (QDs) permit to gather a great part of solar energy in a variety of modes, among which multiple exciton generation [1,6], intermediate band formation [7], or modulation of the solar absorption based on the size tuning due to the quantum confinement effect (QCE) [8]. Actually, confined Si (2- to 5-nm QDs) shows a threshold for light absorption (optical bandgap, *E*_g_^opt ^spanning over 2.0 to 2.8 eV [9,10], well larger than that of bulk Si (1.1 eV) [11]. Since the actual PV module production is largely dominated by Si (mono, poly-crystalline, or amorphous), the enhancement of energy conversion efficiency through Si-based or Si-compatible nanostructures could lead to a breakthrough in the PV market.

Recently, the variation of the Si QD optical bandgap was experimentally shown to rely not only on the size tuning but also on the deposition technique (comparing sputtering and chemical vapor deposition methods) and on the amorphous-crystalline (*a*-*c*) phase of the nanoclusters [10]. Moreover, theoretical calculations confirmed that the amorphization of Si nanoclusters reduces the fundamental gap and increases the absorption strength [12,13]. Some trial PV devices have been fabricated with Si QDs (size of 3 to 8 nm) embedded in SiO_2_, exhibiting a conversion efficiency up to 10% [14]. In similar devices, a poor carrier transport has been evidenced as a limiting factor for cell performance and a maximum open circuit voltage of 410 mV was measured, well below that of single-junction mono-crystalline Si solar cell [15]. Thus, at present, PV cells based on Si QDs do not show encouraging characteristics. On the other hand, passing from bulk to confined Si, *E*_g_^opt ^hops from 1.1 to about 2.0 eV, opening a not-negligible break in the solar energy harvesting by Si. Thus, new nanostructured materials, Si compatible, are required to fill this gap.

Recently, Ge QDs are attracting a larger attention for their potential applications in PV because of the lower fabrication temperature and of the larger excitonic Bohr radius (approximately 20 nm) with respect to Si (approximately 5 nm) [11,16], this allowing in principle an easier modulation of the electronic properties by the QCE. Moreover, since the electronic bandgap of bulk Ge (0.66 eV) is well lower than that of bulk Si (1.1 eV) [11], the QCE in Ge QDs could allow the modulation of *E*_g_^opt ^within the energy range (1.1 to 2.0 eV) where bulk or confined Si fails. Up to now, Ge QDs embedded in SiO_2 _have been widely studied for optoelectronic applications [16-20], with a nearly size-independent photoluminescence which was not attributed to simple confinement effect but probably to the QD/matrix interface [16,19]. Only a few studies have been performed on nanoscaled Ge clusters for PV application, mainly focused on their fabrication within SiO_2 _matrix [21,22], or on the combination with titania nanoparticles [23]. In addition, the sunlight absorption in these nanostructures has been poorly characterized, and a univocal consensus on the underlying mechanism has not been reached.

The absorption spectrum (*α*) of Ge QDs has been experimentally measured, and it was shown that the two main peaks visible in *α *of bulk Ge (i.e., the *E*_1 _and *E*_2 _direct transitions at 2.1 and 4.3 eV, related to the band structure of bulk Ge [24]) disappear by shrinking the QD size below 3 nm, suggesting that the band structure of bulk can be altered by the confinement [25]. Later on, Tognini and co-workers evidenced a relevant blueshift of *E*_2 _(due to the QCE) and a weakening of *E*_1 _with size reduction of Ge QDs embedded in Al_2_O_3 _[26], while Heath et al. concluded that *E*_1 _and *E*_2 _transitions are apparently unaffected by confinement in Ge QDs produced with ultrasonic methods [27]. For PV application, the *E*_g_^opt ^of embedded Ge QDs is a crucial parameter, but experimental measurements are still lacking. Several theoretical studies predict that it increases up to 5 eV by reducing the QD size below 1 nm, while it is fairly constant at a value of 1.5 eV for size larger than 6 nm [28,29].

In order to verify these calculation results and to test the application of Ge QDs for PV, some open questions are whether the size of such nanostructures is the only parameter determining the sunlight absorption and to which extent, and whether there is some effect related to the structural phase (*a *or *c*) of Ge QD or to the QD-matrix interfaces. In this paper, we report an experimental investigation on the photon absorption in Ge QDs (2 to 10 nm in size) embedded in silica, providing the thermal evolution of the absorption spectra in connection with the *a*-*c *transition and the QD ripening. An optical bandgap of 1.6 eV has been found with clear evidence that light absorption is mediated by electronic states localized at the interface between Ge QDs and the hosting matrix.

### Experimental

Ge QDs embedded in silica have been obtained by magnetron co-sputtering of SiO_2 _and Ge targets (Ar atmosphere, nominal deposition temperature 400°C), upon fused silica substrates. Thermal annealing in the 600°C to 800°C range (1 h, N_2 _ambient) promoted the phase separation of SiGeO film into SiO_2_, GeO_2_, and Ge clusters (due to precipitation of the exceding Ge). The thickness of the SiGeO film (approximately 280 nm) was measured by transmission electron microscopy (TEM), and the elemental composition was determined by Rutherford backscattering spectrometry (RBS, 2.0 MeV He^+ ^beam). The spectra, simulated with SIMNRA software [30], revealed that in the as-deposited sample, the Si, Ge, and O contents are 24, 16, and 60 at.%, respectively, homogeneous in depth. Because of the annealing, the overall Ge amount contained in the SiGeO film slightly decreases from 3.0 × 10^17 ^cm^-2 ^(in the as-deposited sample) to 2.6 × 10^17 ^cm^-2 ^(800°C-annealed sample) due to the Ge out-diffusion through the surface, as already evidenced in the literature [20]. Normal transmittance (*T*) and the 20° reflectance (*R*) spectra in the 200- to 2000-nm wavelength range were measured, by using a Varian Cary 500 double beam scanning UV/Visible/NIR spectrophotometer (Agilent Technologies, Inc., Santa Clara, CA, USA) for extracting the absorption coefficient of the films, as described in Ref. [10]. Cross-section transmission electron microscopy in high resolution (HR-TEM) or scanning mode (STEM) was used to verify the formation of Ge clusters, to measure their size distribution, and to evidence the crystalline phase. The observations were carried out using a JEOL 2010F microscope (JEOL Ltd., Tokyo,Japan) operating at 200 kV equipped with a Schottky field-emission gun, a Gatan imaging filter (GIF) for compositional mappings, and a JEOL STEM unit, with an annular dark-field detector operated in high angle (HAADF) mode for Z contrast imaging. In addition, *c*-Ge clusters have been characterized also with glancing-incidence X-ray diffraction (GI-XRD) analysis, using the *K*_α _radiation of Cu (*λ *= 0.154 nm), fixing the incidence angle at 0.5° and performing the 2*θ *scan. Basing on the (111), (110), and (220) Bragg diffraction peaks of the GI-XRD spectra (not shown), the average QD size was estimated by applying the Scherrer formula [31].

## Results and discussion

**Figure 1:**
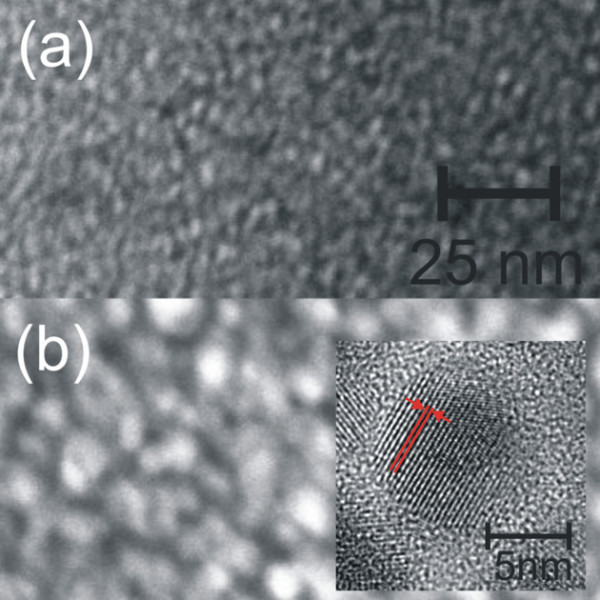
**Cross sectional dark-field STEM images (same magnification) of the sample**. As deposited **(a) **or after annealing at 750°C **(b)**. The inset reports a HR-TEM of the annealed sample, showing the presence of a clear crystalline structure.

**Figure 2:**
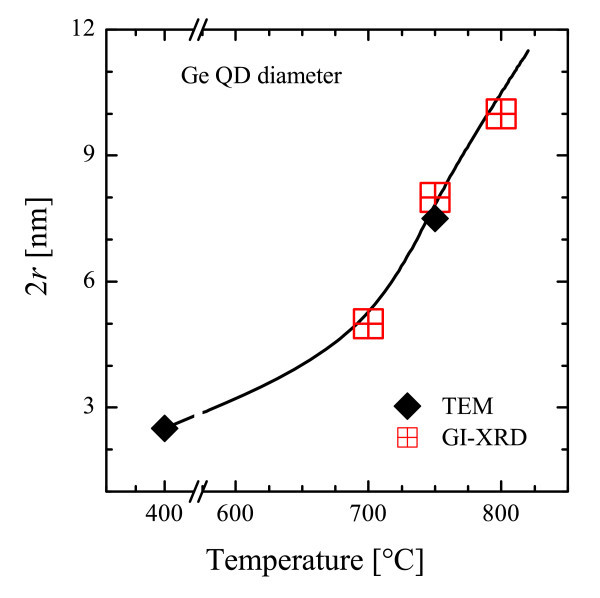
**Thermal evolution of the mean diameter (2*r*) of Ge nanostructures**. Measured by TEM (diamond) or GI-XRD (squares). Line is a guide for eyes (color online).

A high density of Ge precipitates within the SiO_2 _matrix is revealed by the STEM images (at the same magnification) in Figure 1, just after the deposition (a) and after thermal annealing at 750°C (b). The bright patches represent Ge nanoclusters whose density and mean size noticeably change after annealing (the mean diameter increasing from 2.5 to 7.5 nm). Although Ge QDs are already present in the as-deposited films, as recently found also by Zhang et al. [22], the deposition temperature was not high enough to induce the formation of crystalline QDs in our case. SiGeO film deposited by sputtering can be described as a mixture of Ge, GeO_2_, and SiO_2 _units, according to a random matrix model, similarly to what occurs for silicon-rich oxide [32]. During annealing, Ge QDs undergo an Ostwald ripening mechanism, similar to the Si QD case [33], leading to a size increasing of precipitates with a concomitant *a*-*c *transition occurring in the 600°C to 800°C range [20]. The inset in Figure 1b reports an HR-TEM image of the annealed sample, evidencing a clear crystalline phase for Ge QD with the fringes due to crystalline planes (indicated by red lines and separated by 0.33 nm, as the (111) planes of *c*-Ge bulk). In Figure 2, the mean QD diameter (2*r*) measured by TEM (diamond) and by GI-XRD (crossed squares, line is a guide for eyes) is reported as a function of the annealing temperature. Even if GI-XRD gives information only on *c*-QDs, the reasonable agreement between the two techniques observed at 750°C is supporting the idea that the size distribution of *c*-QDs does not significantly deviate from that of *a*-QDs. The overall variation of *r *can be extracted by joining the two techniques, showing a clear QD enlargement in the 400°C to 800°C range compatible with an Ostwald ripening mechanism.

**Figure 3:**
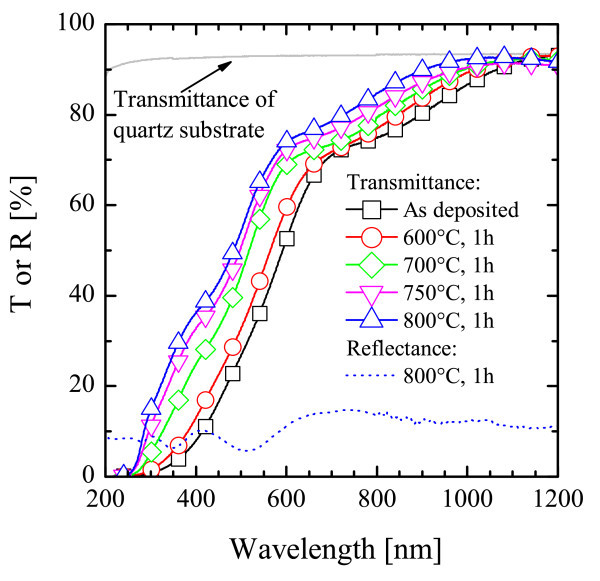
**Transmittance and reflectance spectra**. Transmittance spectra for the bare substrate (quartz, continuous line) and for the as-deposited and annealed SiGeO samples (symbols). The reflectance spectrum (*R*) for the SiGeO sample after annealing at 800°C is also reported (dotted line) (color online).

In Figure 3, the transmittance (*T*) spectra of some SiGeO samples are plotted (symbols) together with that of the quartz substrate (*T *~ 90%, the missing 10% being due to reflection by the quartz surface, not reported here). The presence of Ge QDs induces, in the 200 to 1000 nm range, a strong decrease of *T *which is modulated with the annealing temperature. On the other hand, the reflectance (*R*) spectrum does not depend on the temperature (thus, only the 800°C-annealed sample was reported) and *R *is quite low (approximately 10%) and constant, except for the typical oscillations caused by the beam interference at the air-SiGeO and SiGeO-quartz interfaces. The decrease of *T *for wavelengths smaller than approximately 1000 nm shows the absorption of light related to the presence of Ge QDs embedded in the film. On the other hand, the blueshift of *T *for higher annealing temperatures cannot be straightforwardly related to the Ostwald ripening of Ge QDs, since a redshift should be expected basing on the QCE (the larger QD, the lower the optical bandgap). Thus, the optical transmittance of this SiGeO film is clearly affected by the thermal treatments, but to find a relationship with the structural changes, the absorption spectra should be calculated.

To study the light absorption of these Ge nanostructures, transmittance and reflectance spectra have been used to extract the absorption coefficient (*α*) as follows:

$$\alpha =\frac{1}{d}\ln\frac{T_{\text{Q}}\left(1-R_{\text{S}}\right)}{T_{\text{S}}}$$

where *d*, *T*_S_, and *R*_S _are, respectively, thickness, transmittance and reflectance of the sample, while *T*_Q _is the transmittance of the quartz substrate. The overall indetermination on *α*, also including errors in *d*, *T*, and *R*, has been estimated to be about 5%, while the dynamic range for *α *in our measurements was approximately 1 × 10^3 ^to 2 × 10^5 ^cm^-1^.

**Figure 4:**
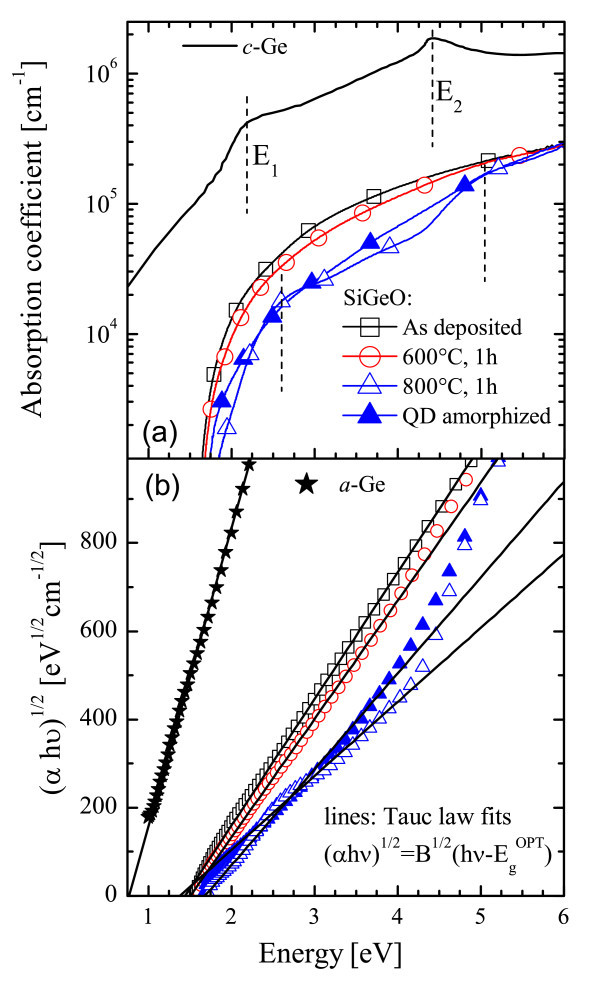
**Absorption spectra, Tauc plots, and relative linear fits**. **(a) **Absorption spectra of SiGeO samples annealed at various temperatures (1 h, N_2 _ambient), together with the spectrum of crystalline Ge [34]. Ion implantation (1.3 × 10^14 ^Ge/cm^2^, 600 keV, max Ge density lower than 0.01 at.%) was performed to induce the amorphization of Ge QDs. **(b) **Tauc plots (symbols) and relative linear fits (according to the reported law, lines) for the same samples and for a thin (120 nm) amorphous Ge film (color online).

Selected *α *spectra are reported in Figure 4a for the as-deposited sample (squares) or after annealing at 600°C (circles) and 800°C (open triangles). The absorption spectrum of crystalline Ge (*c*-Ge, continuous line) is also reported for comparison [34]. The difference of about one order of magnitude between bulk Ge and our sample is not surprising since the main part of the SiGeO film is a transparent matrix (SiO_2 _and GeO_2_), while the Ge involved in QD formation is about 10 at.%. Thus, the reported *α *spectra can be associated to the photon absorption by Ge QDs. Annealing at 600°C does not significantly modify the absorption of Ge QDs, while the change of *α *at 800°C is inferred to the presence of crystalline QDs (evidenced by TEM already at 750°C). In fact, at 800°C, two broad peaks (dashed vertical lines) at about 2.6 and 5 eV appear in the spectrum, recalling the *E*_1 _and *E*_2 _direct transitions (at 2.1 and 4.3 eV) of the bulk *c*-Ge spectrum, but at a slightly larger energy. Such broad peaks in the 800°C-annealed sample can be related to direct transitions within the *c*-Ge QDs having an energy band structure modified by the confinement.

To investigate the role of the QD structural phase, we induced the *c*-*a *transition of the Ge QDs in the sample annealed at 800°C by means of an ion implantation process followed by 550°C, 1-h annealing. The ion implantation parameters (1.3 × 10^14 ^Ge/cm^2^, 600 keV, max Ge concentration lower than 0.01 at.%) were chosen to induce the *c*-*a *transition in a 500-nm-thick *c*-Ge film, which is enough to ensure the full amorphization of our Ge QDs [35]. Post-implant thermal treatment is needed to anneal the matrix damage without inducing re-crystallization of Ge QDs. The absorption spectrum (closed triangles) of the amorphized Ge QDs is reported in Figure 4a. The *c*-*a *transition of Ge QDs does not modify the onset of light absorption neither the spectrum itself, except that for the disappearance of the direct resonance peaks as expected because of the lost crystalline order within the Ge QDs. It should be remarked that the *c*-*a *transition in Si QDs embedded in SiO_2 _actually modifies the absorption by lowering the optical bandgap of about 0.4 eV [10]. This effect has been predicted to occur in both Si and Ge QDs by theoretical calculations of the electronic bandgap [12,13]. Thus, the data presented in this work evidence a divergence in the behavior of Ge QDs with respect to Si ones. Moreover, in Ge QDs, the *α *spectra at 800°C (both *c*- or *a*-Ge QDs) are halved with respect to as-deposited sample, while the Ge content reduction due to Ge out-diffusion was measured to be less than 20%. Thus, annealing at high temperatures clearly induces a not-negligible fall in the light absorption efficiency of Ge QDs, while QD structural phase does not affect the onset of light absorption.

To account for these effects, the Tauc law, describing *α *in amorphous semiconductors, has been used [36]:

$$\alpha =\frac{B}{\text{h}\nu }{\left(\text{h}\nu -E_{\text{g}}^{\text{opt}}\right)}^{2},$$

where hν, *B*, and *E*^opt^_g _are the incoming photon energy, the Tauc constant, and the optical bandgap, respectively. The photon absorption leads to transitions between the extended electronic states from the valence band toward the conduction band, being *E*^opt^_g _the energy difference and *B *proportional to the convolution of the density of electronic states (DOS) in the two energy bands. The Tauc plots, (*α*hν)^1/12 ^versus hν, of selected samples are reported with symbols in Figure 4b, while lines are the linear fit used to determine *B *and *E*^opt^_g_. For reference, a thin (120 nm) amorphous Ge film was deposited on quartz, and its Tauc plot (stars) is also reported with its fit. Tauc plots have a linear slope over a wide range of energy, and the very good agreement between fits and experimental data justifies the Tauc approach.

The optical bandgap of *a*-Ge results 0.8 eV, in good agreement with the literature [37], while the samples containing Ge QDs always exhibit an *E*^opt^_g _of approximately 1.6 eV (well larger than not-confined Ge), independently of the annealing temperature and of the structural phase (*a *or *c*). A similar *E*^opt^_g _has been reported in the literature only for one sample containing Ge QDs in a TiO_2 _matrix [23], without variation of annealing temperature or structural phase. In order to account for the *E*^opt^_g _of QDs, quantum confinement effect can be invoked since the size is well below the excitonic Bohr radius. In Figure 2, the QD size enlargement was reported, but it is not accomplished by a reduction of the *E*^opt^_g_, as expected if only the confinement rule applies. Such a contrast indicates that the confinement rule alone cannot account for the mechanism of photon absorption in Ge QDs, or it is masked by a stronger phenomenon.

**Figure 5:**
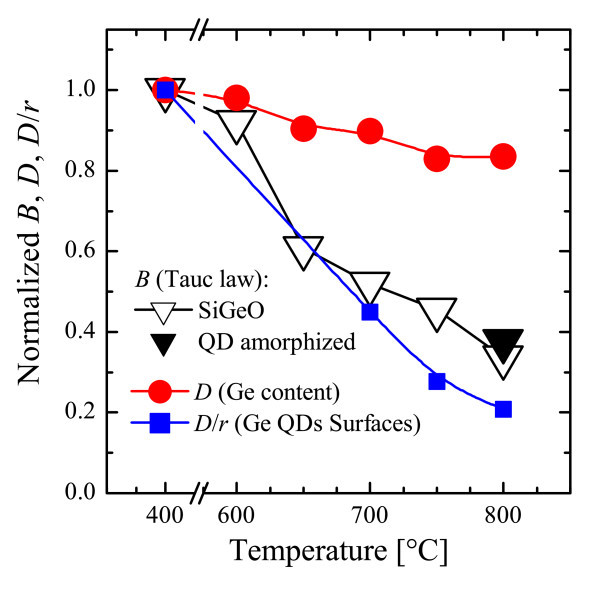
**Tauc constant, Ge content, and the surfaces of Ge QDs**. Comparison between the Tauc constant (*B*, triangles) as obtained from Tauc fits, the Ge content (*D*, circles) as measured by RBS, and the surfaces of Ge QDs (*D*/*r*, squares). All the values have been normalized to that of the as-deposited sample (color online).

The reduction of α with temperature (Figure 4a) can be instead ascribed to a significant decreasing of the Tauc constant (*B*) as evident from the falling slopes of fits in Figure 4b. In fact, the *B *values, normalized to the as-deposited case, are reported as open triangles in Figure 5, revealing that after 800°C annealing, the DOS in Ge QDs involved in the light absorption (proportional to *B*) is strongly reduced to about one third, independently of the Ge QDs phase (*c *or *a*, open or closed triangles, respectively). If the DOS was related only to the density of Ge-Ge bonds, the *B *trend would decrease as much as the Ge content in the film (*D*, circles in Figure 5, as measured by RBS and normalized to the as-deposited case), but this is not the case. Instead, the photon absorption could be related to Ge bonds near the QD surfaces. If so, given a fixed amount of clustered Ge, the *B *value would be larger the smaller is *r*. Since the surface to volume ratio is proportional to 1/*r *and the volume is proportional to *D*, the total area of the surfaces of Ge QDs should decrease as *D/r*, reported in Figure 5 as squares. The patent correlation between *B *and *D/r *trends clearly suggests that the light absorption in Ge QDs embedded in SiO_2 _is strongly influenced by the surface of Ge QDs. In addition, such an evidence can account also for independence of *E*^opt^_g _on the QDs size or phase, since the photon absorption seems to be mediated by surface electronic states, not related to the volume of QDs.

These surface electronic states can be related to the presence of Ge dangling bonds or Ge-O or Ge-Si bonds located near the QD surface, or to the surface itself which induces an atomic rearrangement with different bond angle and bond length than in the bulk. To test the presence of dangling bonds, we annealed some samples (as deposited, or annealed at 700°C or 800°C) in forming gas ambient (Ar/H = 95:5 mixture, 1 h at 450°C) which is known to saturate dangling bonds in disordered structures. The optical *T *and *R *of these samples were unaffected by the forming gas treatment, so we can state that the observed behavior in the light absorption is not influenced by dangling bonds. On the other hand, a strong Fermi-level pinning near the top of valence band in bulk Ge has been recently evidenced, preventing the formation of a reliable *n*-channel MOSFETs device [38-40]. Such an effect was shown to be caused by native defects at the Ge surface, which modify the density of acceptor-like and donor-like states nearby the surface with respect to those in the bulk, and thus largely vary the electronic properties through a significant upwards band bending close to the surface. Actually, surface states in semiconductors typically induce a shift of the charge neutrality level (CNL) towards one of the energy bands. In Si, or in GaP or in GaAs, the CNL at the surface is located above the valence band by about one third of the respective energy bandgap [41], while in Ge it was recently shown to be above the valence band by only one eighth of the bandgap [38-40]. In addition, Schottky barrier heights in metal/Ge contacts are shown to be weakly dependent on the metal work functions [38-40], denouncing a very large density of interface states [39]. Thus, Ge surface largely dominates the electronic properties nearby the surface, much more than in other semiconductors, through a strong pinning of the Fermi level and a significant band bending. Since such a band bending is expected to extend largely for undoped Ge, quantum dots as large as 10 nm can show an overwhelming surface effect on the energy band structure. In this scenario, the expected quantum confinement effect could be masked by the influence of surface states and then the theoretical calculation should reconsider these states for the optical bandgap determination.

## Conclusions

In conclusion, we have produced and characterized Ge QDs (2 to 10 nm in size) embedded in silica by thermal annealing of a SiGeO film produced by magnetron sputtering. The light absorption spectra of the investigated Ge QDs have been measured, demonstrating that the optical bandgap of these nanostructures, both in the amorphous or crystalline phase, is pinned at about 1.6 eV, regardless of the QD size and then of the confinement extent. Moreover, we showed that for a given amount of clustered Ge, the probability of photon absorption is larger the smaller is the QD size. By modeling the photon absorption mechanism, we evidenced that it is related to the surfaces of Ge QDs rather than to their volume, through the mediation of the electronic states localized at the interface between Ge QDs and the hosting matrix. This behavior has been discussed in comparison with the Fermi-level pinning observed in metal/Ge contacts. The reported surface effect on the light absorption in Ge QDs should be kept into account for both the electronic gap calculations and for any application in photovoltaic devices. As far as the optical bandgap is concerned, Ge QDs, in conjunction with confined and bulk Si, give the chance to efficiently modulate the onset of light absorption from 1.1 eV (bulk Si) up to more than 2 eV (Si QDs).

## Competing interests

The authors declare that they have no competing interests.

## Authors' contributions

SC contributed to samples processing, characterization (UV/Visible/NIR and GI-XRD), data analysis and interpretation, and drafted the manuscript. SM conceived the study, contributed to sample characterization (RBS, GI-XRD), data analysis and interpretation, and revisited the manuscript. MM and RLS realized the SiGeO films. GN and CS provided TEM analysis. FS contributed to optical analysis. AT conceived the study, contributed to data interpretation, coordinated the work.

All authors read and approved the final manuscript.
